# Molecular Survey of Bacterial Communities Associated with Bacterial Chondronecrosis with Osteomyelitis (BCO) in Broilers

**DOI:** 10.1371/journal.pone.0124403

**Published:** 2015-04-16

**Authors:** Tieshan Jiang, Rabindra K. Mandal, Robert F. Wideman, Anita Khatiwara, Igal Pevzner, Young Min Kwon

**Affiliations:** 1 Department of Poultry Science, University of Arkansas, Fayetteville, Arkansas, United States of America; 2 Cell and Molecular Biology Program, University of Arkansas, Fayetteville, Arkansas, United States of America; 3 Cobb-Vantress Inc., Siloam Springs, Arkansas, United States of America; Wilfrid Laurier University, CANADA

## Abstract

Bacterial chondronecrosis with osteomyelitis (BCO) is recognized as an important cause of lameness in commercial broiler chickens (meat-type chickens). Relatively little is known about the microbial communities associated with BCO. This study was conducted to increase our understanding of the microbial factors associated with BCO using a culture-independent approach. Using Illumina sequencing of the hyper-variable region V6 in the 16S rRNA gene, we characterized the bacterial communities in 97 femoral or tibial heads from normal and lame broilers carefully selected to represent diverse variations in age, line, lesion type, floor type, clinical status and bone type. Our in-depth survey based on 14 million assembled sequence reads revealed that complex bacterial communities exist in all samples, including macroscopically normal bones from clinically healthy birds. Overall, *Proteobacteria* (mean 90.9%) comprised the most common phylum, followed by *Firmicutes* (6.1%) and *Actinobacteria* (2.6%), accounting for more than 99% of all reads. Statistical analyses demonstrated that there are differences in bacterial communities in different types of bones (femur vs. tibia), lesion types (macroscopically normal femora or tibiae vs. those with pathognomonic BCO lesions), and among individual birds. This analysis also showed that BCO samples overrepresented genera *Staphylococcus*, whose species have been frequently isolated in BCO samples in previous studies. Rarefaction analysis demonstrated the general tendency that increased severities of BCO lesions were associated with reduced species diversity in both femoral and tibial samples when compared to macroscopically normal samples. These observations suggest that certain bacterial subgroups are preferentially selected in association with the development of BCO lesions. Understanding the microbial species associated with BCO will identify opportunities for understanding and modulating the pathogenesis of this form of lameness in broilers.

## Introduction

Generalized leg weakness and lameness are prominent economic and welfare concerns among poultry producers. Lameness in poultry can have several causes including viral and bacterial infections of bone and cartilage. Bacterial chondronecrosis with osteomyelitis (BCO, formerly known as femoral head necrosis, proximal femoral degeneration, or bacterial chondronecrosis, BCN) is recognized as an important cause of lameness in commercial broiler chickens (meat-type chickens). BCO commonly develops in the proximal femur and proximal tibiotarsus (tibia) where the wide, thick growth plates are susceptible to mechanical damage (osteochondrosis) that predisposes the cartilage to secondary bacterial infection. Physiological stress contributes to enhanced bacterial translocation through the gastrointestinal epithelium. Opportunistic bacteria pass through leaky epithelial tight junctions and spread hematogenously to osteochondrotic micro-fractures and clefts in the proximal femoral and tibial growth plates. Bacterial proliferation and the ensuing immunological response generate the necrotic abscesses and voids that are pathognomonic for BCO [1–5]. BCO has been diagnosed in broilers in Australia, Canada, Europe and the US, and is considered the most common cause of lameness in broilers [2,4,6–10]. Multiple opportunistic organisms in mixed cultures have been isolated from BCO lesions, including predominately *Staphylococcus spp*., *Escherichia coli*, and *Enterococcus spp*. [4]. However, no previous attempt has been made to investigate the overall composition and diversity of the bacterial communities associated with BCO.

In the past, methods for identifying pathogens relied overwhelmingly on culture techniques followed by biochemical testing using semi-automated platforms in the clinical laboratory [11]. Culture-independent approaches such as molecular profiling of the 16S rRNA gene sequences provide a powerful technique for comprehensively analyzing the structure and diversity of microbial communities [12]. The 16S rRNA gene sequences contain hyper-variable regions that can provide comprehensive information on bacterial communities, including diversity, composition, and community structure [13]. The development of next generation sequencing, 454 pyrosequencing, SOLID and Illumina platforms, provided a major advance by enabling hundreds of thousands of sequences to be collected from multiple samples. This powerful technique has been used to investigate the composition of complex microbial communities in diverse environments [14–18]. The two platforms that are most commonly used for microbial community analysis are 454 FLX and Illumina. The 454 FLX instrument generates ~1 million reads per instrument run, with the read lengths of 250–400bp, and the Illumina platform produces up to 1.5 billion reads per run, with the read lengths of 50–150 bp. The shorter Illumina reads may reduce phylogenetic resolution, both in terms of picking operational taxonomic units (OTUs) and determining phylogenetic distances between OTUs. The number of base pairs per reads for the Illumina platform can be doubled by the paired-end (PE) approach where each molecule is sequenced from both the 5’ and 3’ ends [19,20]. The regions of 16s rRNA gene, V1-V2 and V4, are longer than 200 bp in length, which cannot be easily covered by the PE overlap. However, the V3 [13] and V6 [21,22] regions can be easily covered by the overlapping PE reads.

One of the hindrance in studying BCO lameness in broilers is its low incidence, which makes it difficult to study BCO lameness in a systematic and reliable manner. Recently, Wideman et al. developed a wire-flooring model in which broilers raised on wire flooring reliably triggers high incidences of BCO consistently without purposefully exposing the birds to pathogenic bacteria [4]. The unstable footing created by wire flooring accelerates the formation of osteochondrotic micro-fractures and clefts in the proximal epiphyseal-physeal growth plates of the femora and tibiae. Wire flooring (or the lack of access to litter) also triggers physiological stress that can lead to immunosuppression and enhanced bacterial translocation from the gastrointestinal tract into the systemic circulation [3–5,23].

In this study, we used the Illumina paired-end sequencing method targeting V6 hyper-variable region of 16S rRNA gene in conjunction with wire flooring model of BCO lameness for comprehensive investigation of the bacterial communities associated with BCO.

## Materials and Methods

### Animal experiment and sample collection

Animal procedures were approved by the University of Arkansas Institutional Animal Care and Use Committee. For the present study we used environmental chambers 7 through 12 within the Poultry Environmental Research Laboratory at the University of Arkansas Poultry Research Farm. These chambers (3.7 × 2.5 × 2.5 m, length × width × height) utilize single-pass ventilation at a constant rate of 6 m^3^ per minute per chamber. Chambers 7 and 8 were set up with 3.05 m x 1.5 m pens having wood shavings litter flooring to serve as the baseline (non-challenge) control. Chambers 9 through 12 were set up with 3.05 m x 1.5 m pens and flat wire flooring wire panels. The wire flooring panels were constructed from 5 cm x 5 cm lumber and were 3 m long and 1.5 m wide, with 5 cm x 5 cm cross members added for support. Hardware cloth (1.3 cm x 2.54 cm mesh = 0.5 inch by 1 inch, 0.063 gauge, galvanized welded wire cloth) was fastened to the top of the frame and cross-members. The wire flooring was elevated on 30 cm high masonry blocks to permit manure to pass through and accumulate underneath the wire surface. Tube feeders were positioned at the front and nipple waterers were positioned at the rear of each pen, thereby forcing the chicks to traverse the length of the floor to eat and then drink [4]. The photoperiod was set for 23 h light:1 h dark throughout the experiment. Thermoneutral temperatures were maintained throughout, with target temperatures set at 32°C for d 1 to 3, 30°C for d 4 to 6, 28°C for d 7 to 10, 26°C for d 11 to 14, and 24°C thereafter. Feed and water were provided ad libitum. The diets were corn and soybean meal-based broiler starter (crumbles) and finisher (pellets) feeds formulated to meet minimum NRC [24] standards for all ingredients. Chicks from two commercial lines (B and D) were obtained from a commercial hatchery and were wing banded on the day of hatch. Line B is known to be more susceptible to BCO than Line D (Wideman, personal observations). All chicks initially were housed on litter flooring, with equal numbers of birds from the two lines comingled in chambers 7 and 8 (130 birds per line per chamber). On days 7 and 14, five birds per line were randomly selected and removed for analysis of bacterial communities from chambers 7 and 8 (n = 10 per line on days 7 and 14). On day 14 the remaining chicks in chambers 7 and 8 (120 birds per line per chamber) were redistributed evenly among all 6 chambers (2 lines per chamber; 40 birds per line per chamber). Thereafter at least five birds per line were randomly selected from all six chambers on days 21, 29, 36, 43 and 49 (n = 60 per line per sampling day: 20 from litter flooring and 40 from wire flooring). The selected birds were humanely euthanized by CO_2_ inhalation and, within 30 min post-mortem, the proximal femora and tibiae of both legs were exposed in a laminar flow hood for scoring based on macroscopic lesion appearance (see the following section) and sampling for bacterial community analysis. Forceps and shears that were dipped in 95% ethanol and flame sterilized were used to aseptically remove the upper metaphysis and physis (growth plate) of each proximal bone end. Forceps and shears were re-sterilized immediately before collecting each bone sample. The cut surfaces of the proximal femoral and tibial samples were dipped in 95% ethanol, flame sterilized, dropped into sterile culture tubes, and stored at -20°C for subsequent DNA isolation.

Birds in all chambers were “walked” and observed for lameness every two days beginning on day 15. Birds that developed clinical lameness were humanely euthanized and necropsied. Necropsy observations on dead and lame birds were recorded by day/date, gender and pen number. On day 56 all surviving birds were euthanized, weighed and necropsied. A total of 1,372 bone samples were collected from 343 broilers (4 samples per bird: right and left femora and tibiae) over the course of this experiment. From this massive sample reservoir, 97 individual samples were carefully selected for analysis, representing all 6 diagnostic categories [normal proximal femoral head (Normal Femur); proximal femoral head separation or epiphyseolysis (FHS); proximal femoral head necrosis (FHN); normal proximal tibia head (Normal Tibia); tibial head necrosis (THN); tibial head necrosis severe or caseous (THNsc)], all 7 ages, both flooring types (litter vs. wire), both genetic lines, and both body parts (right vs. left) (S1 Table).

### Macroscopic classification of lesions

**Fig 1:**
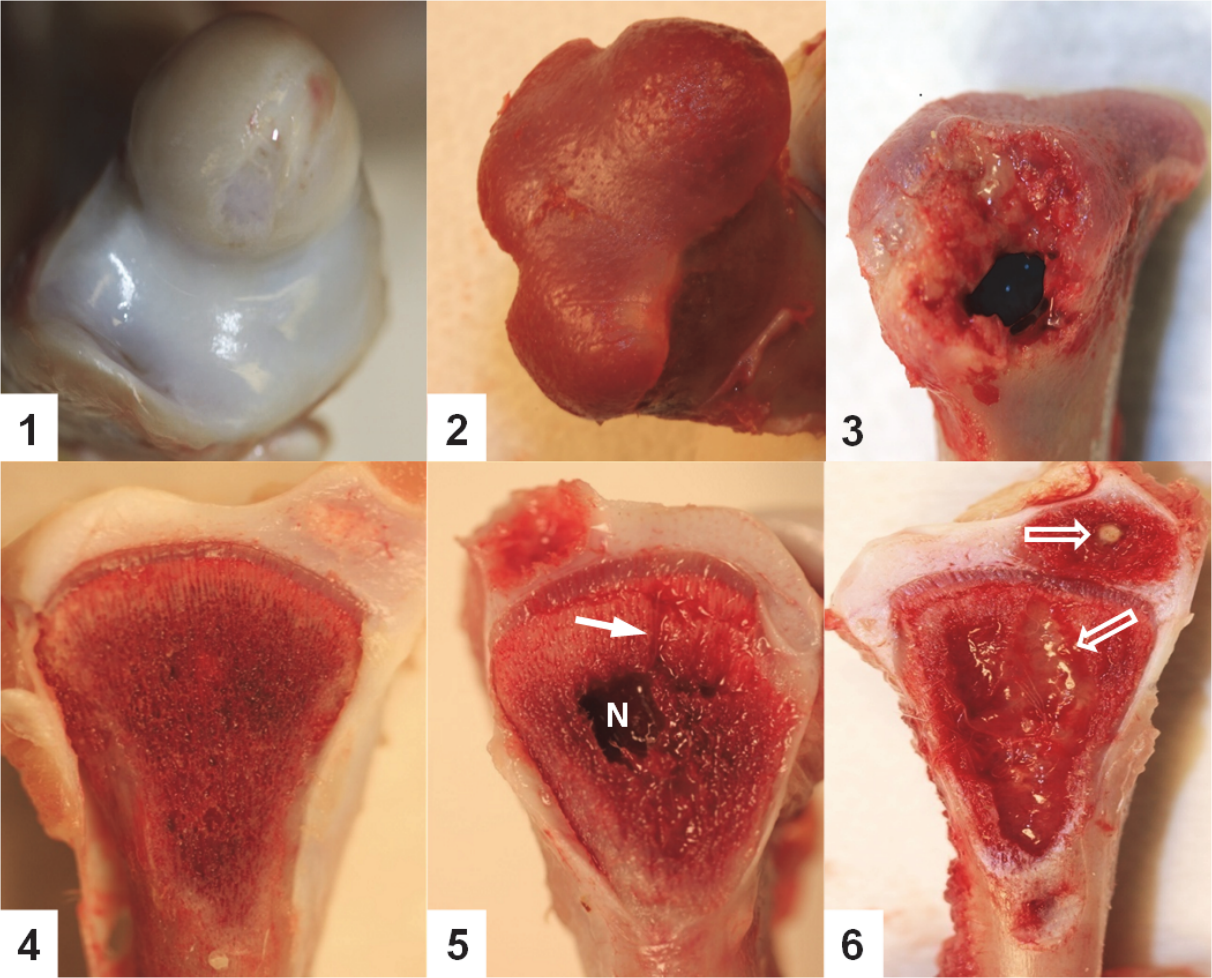
Stages of proximal femoral head degeneration (Upper row) or tibial head necrosis (Low row) leading progressively to bacterial chondronecrosis with osteomyelitis (BCO). 1. Normal proximal femoral head; 2. Femoral head separation (FHS: epiphyseolysis); 3. Femoral head necrosis, FHN. 4. Normal proximal tibial head with struts of trabecular bone in the metaphyseal zone fully supporting the growth plate; 5. Tibial head necrosis (THN). Lytic channels (small arrows) penetrate from the necrotic voids into the growth plate. 6. Tibial head necrosis (THNsc). Bacterial infiltration and sequestrae (open arrows) provide macroscopic evidence of osteomyelitis.

The femora and tibiae were categorized by macroscopic appearance according to the following diagnostic categories: Normal Femur; FHS; FHN; Normal Tibia; THN; THNsc. In all experiments the proximal femoral head lesions were categorized separately (FHS, or FHN), and the two levels of tibial head necrosis were categorized separately (THN, or THNsc) to emphasize the progressive severity of the BCO lesions [4], as represented in Fig 1.

### DNA isolation, PCR amplification, and amplicon purification

**Fig 2:**
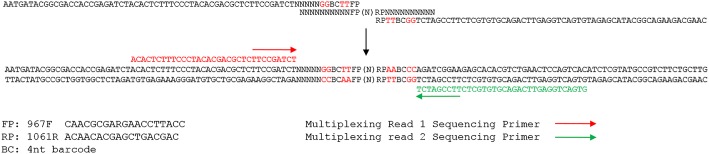
Design of the PCR primers and the amplicon library. V6 rRNA region were amplified using the two primers with barcode tags and Illumina adaptors attached at 5’ ends.

For isolation of bacterial DNA, PBS buffer was added to each sample for 10X dilution, and the samples were homogenized using a tissue tearor. Two hundred microliter of each sample was transferred into a microcentrifuge tube for DNA isolation. DNA was extracted from this cell suspension using a QIAamp DNA Mini kit (Qiagen, Valencia, CA., USA). DNA concentrations were estimated spectrophotometrically using NanoDrop (Thermoscientific, USA). Because of its taxonomic resolution [25], conserved flanking regions [26], and length [21], the hyper-variable region 6 (V6) of the bacterial 16S rRNA gene was selected for this study. PCR amplification was carried out for each sample using Illumina paired-end primers in combination with unique barcode sequence at the 5’ end of each primer (S2 Table). Gloor et al. used paired-end sequencing in combination with unique barcode sequence tags at the 5′ end of each primer to perform microbiome analysis of the V6 region of 272 clinical samples using the Illumina sequencing technology [21]. We modified the primer design to use a different barcodes and added the random 5 nt sequence “NNNNN” in the first 5 nt positions of the forward reads of Illumina sequencing to facilitate more efficient and accurate analysis of clusters during processing of image data [27,28] (Fig 2). The PCRreactionss consisted of approximately 0.1 μg of purified genomic DNA, 1× cloned *Pfu* polymerase buffer, 5 U *Pfu* polymerase (Stratagene La Jolla, CA, USA), 1 mM dNTPs (GE Healthcare Bio-Sciences Corp., Piscataway, NJ), 1.2 μM each primer in a total volume of 50 μL. The DNA engine thermal cycler (Bio-Rad, Hercules, CA, USA) was used with the following amplification conditions: 94°C for 2 minutes; 30 cycles of 94°C sec for 30 sec, 58°C for 60 sec, 72°C for 90 sec; and 72°C for 10 minutes for final extension. PCR products of the correct size were gel-purified (Qiagen, Valencia, CA, USA). PCR products of each sample were mixed in equal quantities, based on measurement with Qubit 2.0 Fluorometer. Illumina sequencing was performed for 100 cycles in paired-end mode with Illumina HiSeq 2000.

### Data Analysis

Illumina reads were binned according to barcode sequences using DNAStar software (DNASTAR, Inc). Overlapping regions within paired-end reads were then aligned to generate assembled reads. If a mismatch was discovered, the paired-end sequences involved in the assembly were discarded. The sequence reads with any ambiguous base calls or without a perfectly matching barcode were also discarded. Then the compiled dataset was analyzed with the software package Quantitative Insights Into Microbial Ecology (QIIME; http://qiime.sourceforge.net)[29] using default settings with one modification: the length cutoff was set at 130 instead of the default 150 bases, as the V6 region was expected to produce amplified fragments between 110 and 130 bp [21]. To match the input file format for QIIME, the assembled sequence reads were modified using a custom Python script.

For Operational taxonomic unit (OUT) analysis, we used the open-reference OTU picking method in QIIME with OTU definition at a level of 97% sequence similarity and Greengenes reference database (February 4th 2012) [30] as a reference sequence collection. Rarefaction curves, alpha (α) diversity and beta (β) diversity calculations were performed and plotted using QIIME. Similarity and differences in community membership were calculated based on the phylogenetic information within each sample with both the unweighted and weighted UniFrac analysis. The analysis of similarities (ANOSIM) [31] function in QIIME was used to test for differences in community composition among groups of samples. UPGMA trees of weighted and unweighted UniFrac values were calculated with QIIME and plotted with FigTree v 1.4.0 (http://tree.bio.ed.ac.uk/software/figtree/). Principal coordinate analysis (PCoA) of weighted and unweighted UniFrac was also performed and plotted with QIIME [29]. LEfSe analysis (linear discriminant analysis effect size) was applied to discover the biomarkers related to BCO [32].

### Statistical analysis

For comparisons of lameness incidences the individual bird was used as the experimental unit, and the SigmaStat Z-test procedure was used to compare proportions (Jandel Scientific, 1994). For comparison of α diversity expressed in Chao1 index, ANOVA was used to determine statistical difference and Duncan analysis was used for separation of the means showing statistical difference. Statistical difference was considered significant at the level of p≤0.05.

## Results and Discussion

**Fig 3:**
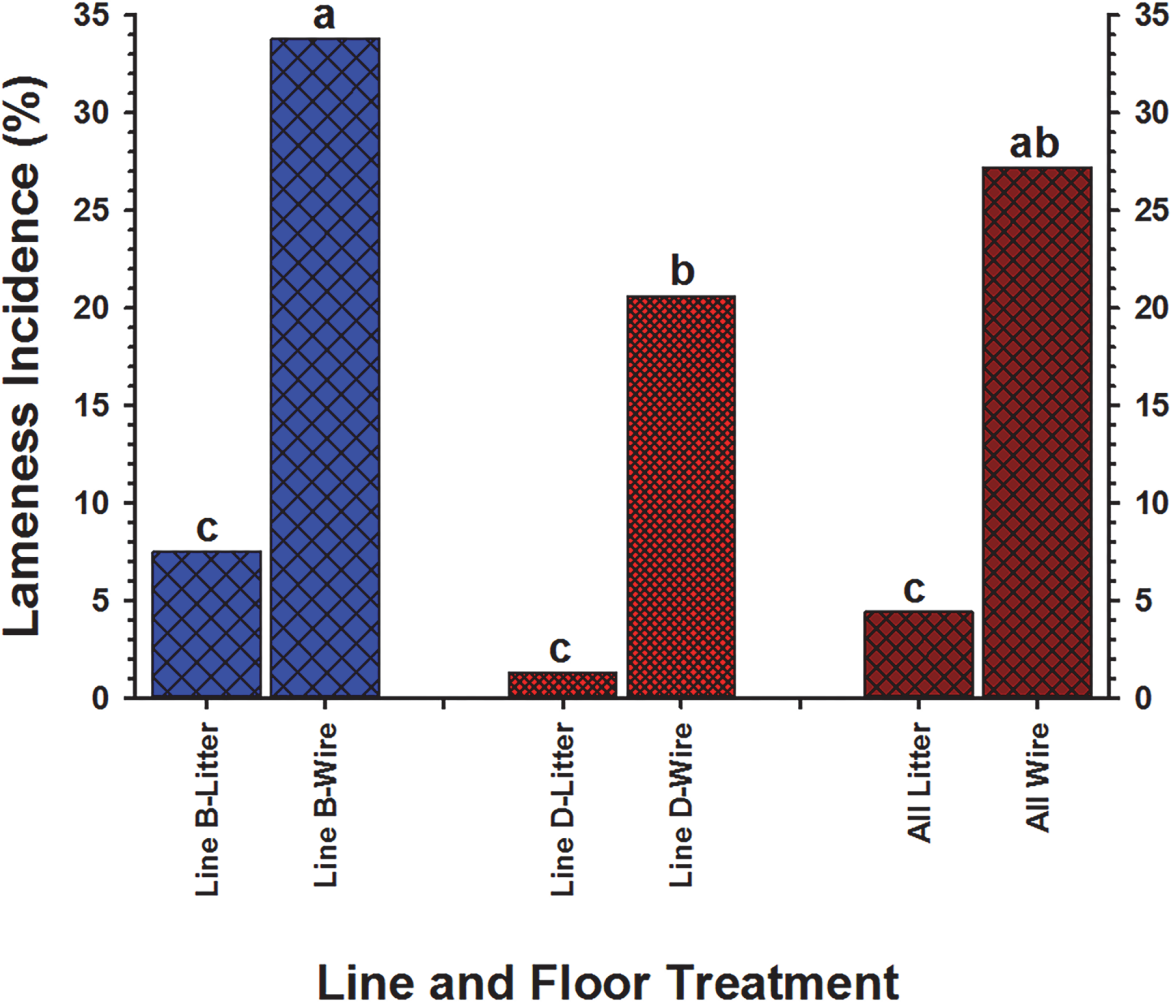
The incidence of lameness in two lines (line B and line D) developed on wire flooring and on wood-shavings litter flooring from days 14 through 49.

BCO is recognized as an important cause of lameness in rapidly growing broilers. BCO appears to be initiated by osteochondrotic micro-trauma to poorly mineralized columns of cartilage cells in the metaphyses and proximal growth plates of the leg bones, followed by colonization by hematogenously distributed opportunistic bacteria [4,5]. Bacteria that translocate into the chick's circulation can spread hematogenously and exit the bloodstream through the fenestrated capillaries supplying the growth plate. Once the osteochondrotic clefts and micro-fractures become infected, leukocytes migrate into the infected area, and, in their attempt to engulf the infectious organisms, release enzymes that lyse the bone. Pus spreads into the bone's blood vessels, impairing their flow, and areas of devitalized infected bone, known as *sequestra*, form the basis of a chronic infection. Terminal BCO presents as necrotic degeneration and bacterial infection primarily within the proximal head of the femora and tibiae, as well as in the growth plates of the flexible thoracic vertebrae [2,5]. Rearing broilers on wire flooring facilitates our ability to investigate the pathogenesis of BCO by consistently triggering substantially higher incidences than typically observed when broilers are reared on wood-shavings litter flooring [4]. In the present study significantly more broilers developed BCO lameness on wire flooring than on litter (27.2% vs. 4.4%, respectively; P = 0.002, z-test). More broilers from Line B developed lameness on wire flooring than broilers from Line D (33.8% vs. 20.6%, respectively; P = 0.011, z-test), whereas the lameness incidences for the two lines did not differ on litter flooring (7.5% vs. 1.3%, respectively; P = 0.126, z-test) (Fig 3).

High incidences of BCO are accompanied by the progressive development of pathognomonic lesions even in broilers that are not yet clinically lame [4,5]. Accordingly, we used the wire flooring model to enhance our ability to acquire suitable numbers of diverse bone samples for investigating the bacterial communities associated with BCO lesion progression. Using routine culture methods, multiple opportunistic organisms have been isolated from BCO lesions, including predominately *Staphylococcus spp*., *Escherichia coli*, and *Enterococcus spp*., often in mixed cultures with other microbes including *Salmonella* spp. [4]. However, our understanding of the bacteria present in the BCO lesions based on routine culture methods is limited, because non-culturable bacterial species that constitute a major portion of the bacterial communities remain undetected. With the development of the next generation sequencing technology, it is possible to comprehensively characterize bacterial communities by deep profiling of their 16S rRNA gene sequences. Recent studies have revealed the composition and diversity of the bacterial community in diverse environments, such as the sea [33], soil [34,35], chicken ceca [36], and salivary, fecal or nasal swabs from humans [37–39]. To our knowledge the present study is the first to evaluate microbial communities of non-mucosal tissue samples from animals. Additionally, BCO in broilers provides an excellent animal model for conducting translational research focused on developing effective prophylactic and therapeutic treatments that are relevant to osteomyelitis in young growing human children [5].

### Data processing

By using combinatorial sequence-tagged PCR, we analyzed 97 bone samples by a single lane of Illumina paired-end sequencing run. We obtained 52,684,589 paired-end reads of exactly 100 nucleotides in length from each end before initial quality filtering. After the assembly using DNAStar, there were 15,598,270 assembled reads for the subsequent analysis. Using a custom Python script, the forward and reverse barcodes were extracted from the assembled reads, and combined together to generate new unique barcode for every read from each sample. The assembled reads were then sorted by the combinatorial barcodes and the file format was modified to be compatible for analysis using QIIME. After the quality filtering, 15,077,583 reads were retained for QIIME analysis. After an additional quality filtering, 1,040,076 short reads and 1,489 reads containing ambiguous nucleotides were discarded. The total number of input reads was 14,036,018 and the median sequence length of the assembled reads was 107 bp.

It is important to note that only 29.6% of the raw data were retained after assembly. This occurred because the length of V6 region is 110–130 and the paired end reads of 100 nucleotides are overlapped for almost their entire length. With this long overlapping region, a large portion of the paired reads with any mismatch between the two reads were discarded during assembly. Although the assembly under this high stringency reduced the number of the final assembled reads, it greatly increased the accuracy of the final assembled sequence reads used for downstream analysis.

### Taxonomic analysis

**Fig 4:**
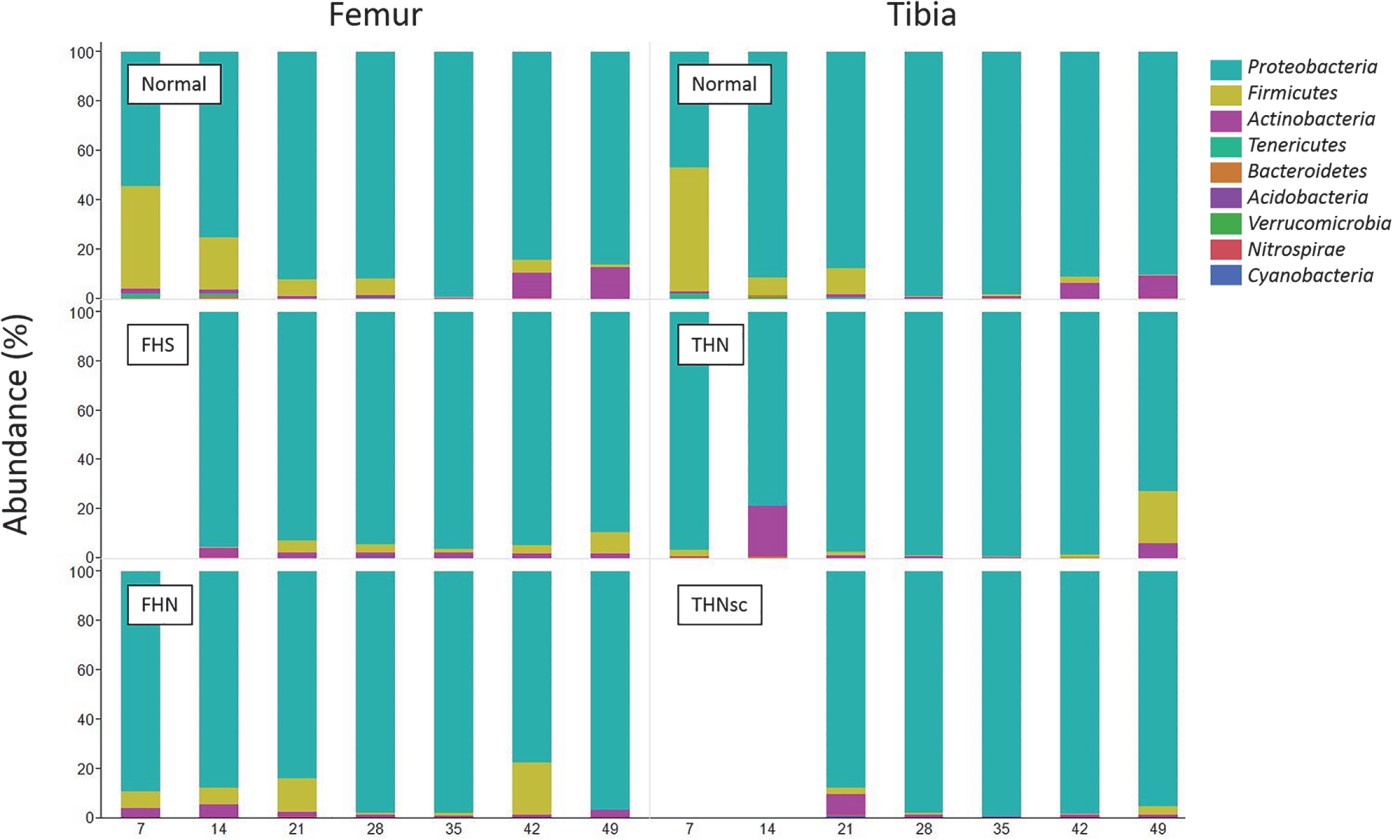
Taxonomic composition of the bone samples at phylum level.

Taxonomic analysis of the 16S rRNA gene V6 region amplicon reads yielded a total of 9 phyla across all samples with the dominant phylum being *Proteobacteria* (mean 90.9%) followed by *Firmicutes* (6.1%) and *Actinobacteria* (2.6%), together accounting for nearly 99.6% of all reads. Several other phyla, including *Tenericutes*, *Bacteroidetes*, *Acidobacteria*, *Verrucomicrobia*, *Nitrospirae* and *Cyanobacteria*, were detected at very low levels (less than 0.4% of the total), nevertheless these phyla were present in multiple samples (Fig 4). Sequence reads also were analyzed on the class, order, family, and genus levels using the QIIME pipeline. The dominant class in all of the bone samples was *Gammaproteobacteria (90*.*5%)*, followed by *Clostridia (3*.*5%)*, *Bacilli* (2.6%) and *Actinobacteria (2*.*5%)*. The dominant order was *Enterobacteriales (34*.*2%)*, followed by *Vibrionales (27*.*3%)*, *Pseudomonadales* (14.6%), *Oceanospirillales* (7.9%), *Pasteurellaceae* (4.7%), *Clostridiales* (3.5%), Actinobacteriales (2.4%), *Bacillales* (1.4%), and *Lactobacillales* (1.1%) (S3 Table).

Bacterial pathogens associated with BCO have been the focus of numerous studies. Most of these previous studies were based on traditional culturing methods. It is believed that BCO is most commonly caused by *Staphylococcus aureus*, but *Escherichia coli*, coagulase-negative staphylococci and *Enterococcus* spp. also have been identified [2,4]. However, not all cases of BCO have been associated with the presence of isolated bacteria [40–42]. Recently two studies used 16S rRNA technology to identify bacteria isolated by culturing infected bone samples [43,44]. Our results show that the bacterial communities in BCO samples are much more complex than previously reported or anticipated. The bacteria observed in the present study include most of the bacteria found in previous studies (S4 Table) at the phylum, class, order, family and genus levels.

### Normal samples

Normal samples included proximal femoral and tibial heads that appeared macroscopically to be normal (Fig 1). A total of 28 normal samples (S1 Table) were collected during weeks 1–7, which includes 4 normal samples per week. The results of taxonomic analysis of the 16S rRNA gene V6 region amplicon reads from normal samples show that there were a total of 9 phyla across all normal samples with the dominant phylum being *Proteobacteria* (mean 84.7%), followed by *Firmicutes* (11.1%) and *Actinobacteria* (3.4%), accounting for nearly 99.2% of all normal samples reads (Fig 4). These results suggest that macroscopically normal bones from broilers that appeared to be clinically healthy are not “sterile” as traditionally assumed. Accordingly, the seemingly normal growth plates and metaphyses of broilers appear to archive multiple bacterial species that reached the bones probably through translocation and hematogenous distribution. Several plausible alternative explanations for this observation must be considered. First, since lesions are visible to the naked eye in only 40 to 67% of the BCO legs necropsied, histological examination has been recommended when no lesions are visible macroscopically. Because histological examination is often not carried out (as in the present study), BCO is almost certainly underdiagnosed [2]. Second, more than 99% of bacterial strains in the environments are resistant to cultivation in the laboratory and therefore would not previously have been identified without culture-independent method such 16S rRNA gene profling. Finally, the 16S rRNA gene profiling method is sensitive, but it cannot distinguish between live and dead bacteria.

There were general trends found in the normal bone samples that were very similar in both femur and tibia: the *Proteobacteria*, the most abundant phylum, increased gradually between 7 and 35 days of age, and then decreased from 35 to 49 days; *Firmicutes* decreased from 7 to 35 days of age; and *Actinobacteria* increased from 35 to 49 days of age in the normal bone samples (Fig 4). The prevalence of *Proteobacteria* in our normal bone samples contrasts with the report by Danzeisen et al. [36](2011) that *Firmicutes* comprise the dominant phylum of the chicken cecal microbiome. The cecum is located at the beginning of the large intestine, whose environments differ greatly from the other body sites, characterized primarily by interaction with the immune system, oxidative challenge, and hydration [45]. With regard to the intestinal tract as a potential site of bacterial translocation, this may suggest that the *Proteobacteria* may translocate more easily than *Firmicutes* through the gastrointestinal epithelium, or survive better than *Firmicutes* inside the blood stream or bones. Beside the gastrointestinal tract, the bacteria also can translocate across the integument or respiratory epithelium and spread hematogenously to the growth plates of the proximal femora and tibiae. Accordingly, translocation via the integument and respiratory system might also account for the differences in microbial communities between the bone and cecal samples.

### Analysis of α and β diversities

**Fig 5:**
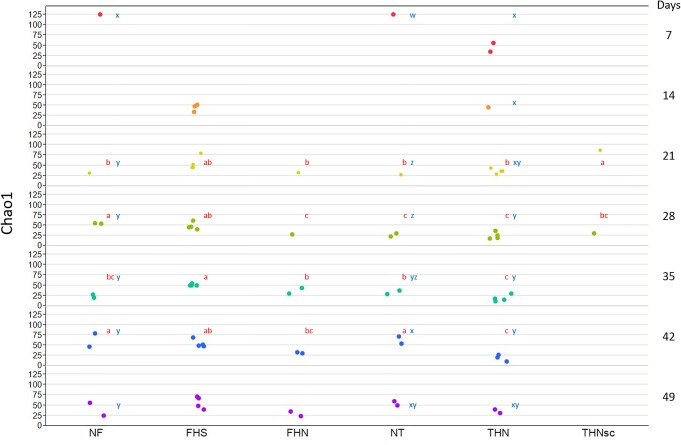
Comparison of the α-diversity in different groups of bone samples. Diversity levels were estimated using Chao1 index with rarefied 180 V6 rRNA reads per sample. Means with the same letters in red color within the same row are not different significantly (p>0.05). Means with the same letters in blue within the same column are not different significantly (p>0.05).

Interestingly, there was a general trend that the diversity within a bacterial community (α diversity) was highest during the first week, and decreased with aging (Fig 5). The opposite trend was reported for chicken cecal samples [36]. These observations suggest selection processes are operating in favor of certain bacterial subgroups in bones in association with aging. The pathogenesis of progressive BCO lesion development, including increases in the size and caseous nature of the most severe bacterial sequestrae, undoubtedly would affect the diversity of the bacterial community, and would be expected to result in decreased diversity with aging. Indeed, a time-course analysis of lesion development demonstrates progressively greater incidences of the early macroscopic FHS and THN lesions over the seven week course of the present experiment. Age-related increases in incidences of the most severe lesions, FHN and THNsc, were less dramatic, likely because birds with BCO lesions in these categories rapidly succumbed to clinical lameness. In addition, the bones with more severe macroscopical lesions were generally associated with reduced diversity of the bacterial communities (Fig 5).

**Fig 6:**
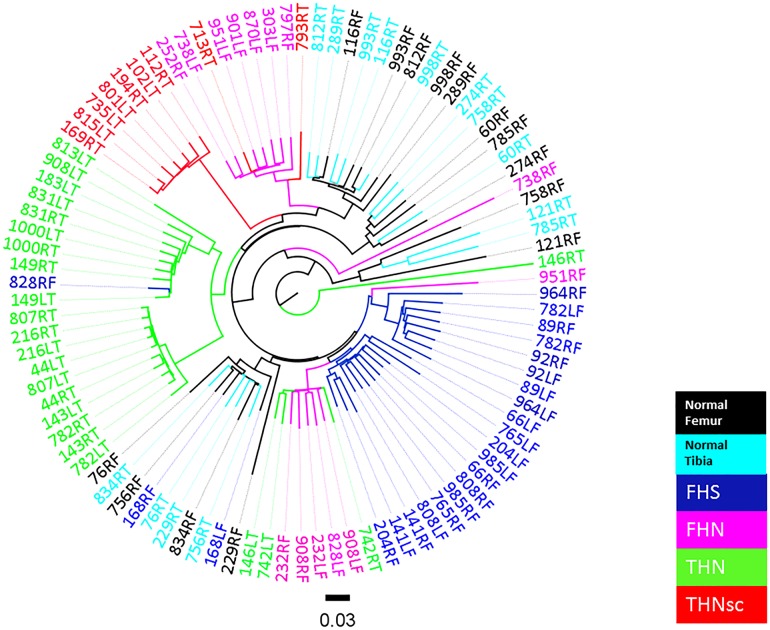
Hierarchical clustering analysis of the bacterial communities. Phylogenetic tree shows that most of FHS (dark blue), THN (green), FHN (pink) and THNsc (red) samples can be clustered. Femur Normal (black), and Tibia Normal (light blue) were largely mixed together in two different clusters.

Hierarchical clustering of the bacterial communities in our 6 BCO diagnostic categories (Normal Femur, FHS, FHN, Normal Tibia, THN and THNsc) was conducted by using the default β diversity metrics of un-weighted UniFrac. The resulting phylogenetic tree shows that most of the FHS, THN, FHN and THNsc samples tended to cluster together within a diagnostic category (Fig 6). The same phylogenetic tree also demonstrates that the Normal femur and Normal tibia samples grouped together in two different clusters that essentially were segregated from the BCO lesion samples. Evidently no distinctive difference existed between these “normal” sample groups from the two different bone types (femur and tibia) (p>0.05).

### Comparison of microbial communities in different groups

ANOSIM analyses (Table 1) demonstrated the presence of distinctively different bacterial communities in different bones (femur vs. tibia, p≤0.001), different lesion categories (normal, FHS, FHN, THN, and THNsc, p≤0.001), different individuals (p≤0.001), and different ages (p≤0.005). However bacterial communities were not different between the samples from right vs. left legs (p = 0.156), between clinically healthy vs. lame broilers (p = 0.714), between broilers reared on litter vs. wire flooring (p = 0.248), or between different lines (Line B vs. Line D; p = 0.089). The lack of different communities between the two lines is important to note because the fact that broilers from Line B are more susceptible than broilers from Line D in developing BCO lameness on wire flooring cannot be attributed to the presence of (or harboring of) distinctively different bacterial communities. Instead other innate factors must contribute to the reproducible differences between these genetic lines in their relative susceptibilities to BCO lameness.

**Table 1:** Analysis of similarities (ANOSIM).

Groups	R-value	P-value
Left vs. Right Leg	0.033512634	0.156
Femoral vs. Tibial Samples	0.216799541	0.001*
Clinically Healthy vs. Lame	-0.041557977	0.714
Individuals	0.626107125	0.001*
Age (7 through 49 days)	0.090237558	0.003*
Litter vs. Wire Flooring	0.02794451	0.248
Lesion Type (normal femur, normal tibia, FHS, FHN, THN, THNsc)	0.4393	0.001*
Line B vs. Line D	0.029131713	0.089

*significantly different (p≤0.05).

**Fig 7:**
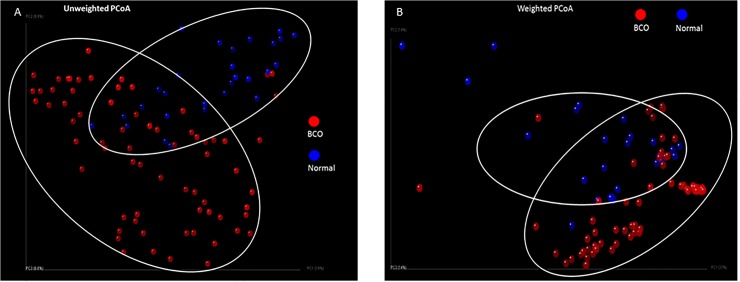
Principal Coordinate analysis. Unweighted PCoA on Qualitative measures and weighted PCoA on Quantitative measures illustrates the differences between Normal and BCO communities. Normal communities are tightly clustered as compared to the scattered BCO communities.

**Fig 8:**
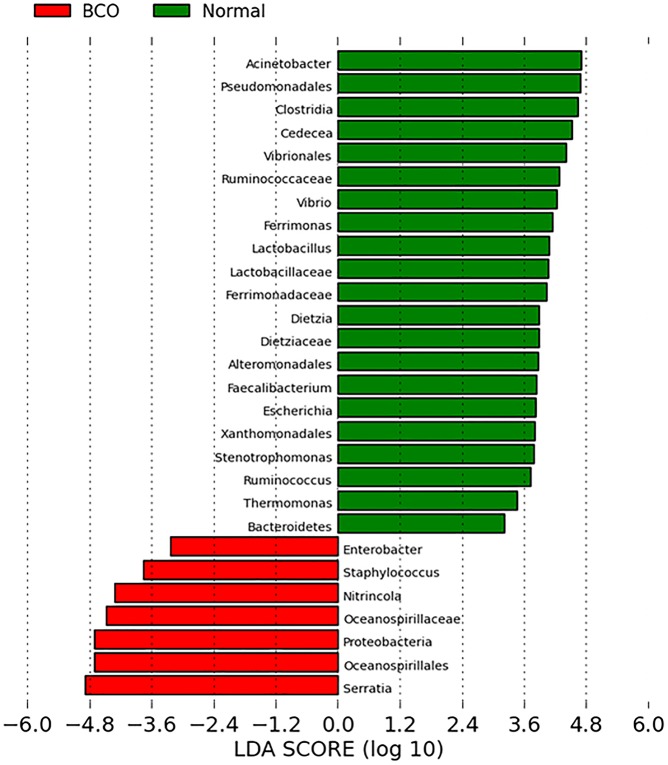
Comparison between Normal and BCO samples using LEfSe analysis. Taxa abundance analysis determined 7 features were differentially abundant in BCO group (LDA score >2.4) and 21 features (LDA >2.4) in Normal cases. The genus *Staphylococcus*, which has been frequently isolated from BCO samples by previous researchers, is an abundant feature in BCO case.

We selected 28 macroscopically normal samples and 69 samples with BCO lesions for analysis, including 23 FHS, 14 FHN, 23 THN and 9 THNsc samples (S1 Table). Principal coordinate analysis (PCoA) shown in Fig 7 visually illustrates the differences between the bacterial communities in these bone samples. The communities in macroscopically normal bones are more tightly clustered as compared to the dispersed communities in bones with BCO lesions. On the other hand, the LEfSe analysis of the communities from Normal and BCO samples shows that there are 28 differentially abundant taxonomic clades with an LDA score higher than 2.4 (Fig 8). The most differentially abundant bacterial taxa in BCO samples belong to phyla: *Proteobacteria*. The genera overrepresented in BCO samples include *Staphylococcus*, *Enterobacter*, *Serrotia* and *Nitrincola*. Interestingly, the genus *Staphylococcus* was detected repeatedly in BCO samples in previous studies using culture-dependent methods [2,8,41,46,47]. The result from our parallel study based on culturing method also showed that in most cases BCO was attributable to *Staphylococcus* species (data not shown). The *Staphylococcus* genus includes at least 40 species. Of these, nine have two subspecies and one has three subspecies [48]. One of the species, *Staphylococcus aureus*, is the most prominent musculoskeletal pathogen of men and animals. It has a particular propensity to infect tissues of the musculoskeletal system, as evidenced by the fact that it is the single leading cause of osteomyelitis in humans [49]. According to ultrastructural studies, adhesion of *S*. *aureus* on damaged growth plate cartilage plays an important role for infection and proliferation within a thick adherent glycocalyx. This situation allows the presentation of a fibrous exopolysaccharide bacterial cell surface to the defense mechanisms of the host and provides a thick barrier to the penetration of antibiotics [50]. Our recent study based on culture independent method indicated that the genus *Staphylococcus is* present in the blood samples of BCO birds (unpublished data). This observation supports the hypothesis that *Staphyloccous* cells enter the blood stream through translocation from gastrointestinal tract and transmitted to bones hematogenously. However, with prevalent nature of *Staphylococcus* on chicken feather and poultry litter [51], it may be possible that *Staphylococcus* enters into the bones through external infection through necrotic lesions on the bone surface.

The remaining three genera that were overrepresented in BCO samples, *Enterobacter*, *Serrotia*, and *Nitrincola*, have not been detected by culture-dependent methods. The genus *Nitrincola* is not common and is not well understood. It is a novel alkaliphilic bacterium isolated from an alkaline, saline lake [52]. Both of the genera, *Enterobacter* and *Serrotia*, are Gram-negative, facultative anaerobic, rod-shaped bacteria of the family Enterobacteriaceae. Several strains of *Enterobacter* are pathogenic and cause opportunistic infections in immunocompromised (usually hospitalized) hosts, and in patients who are on mechanical ventilation. The urinary and respiratory tracts are the most common sites of infection [53]. In the genus *Serratia*, the most common species, *S*. *marcescens*, is recognized as a pathogen and usually causes nosocomial infections. Biofilm formation, and the adherence of microbes to surfaces such as contact lenses, likely plays a role in *S*. *marcescens* pathogenesis [54]. However, rare strains of *S*. *plymuthica*, *S*. *liquefaciens*, *S*. *rubidaea*, and *S*. *odoriferae* have caused diseases through infection [55–58]. These two genera, *Enterobacter* and *Serrotia*, might be important for development of BCO. However, the other three previously reported genera that have been isolated by culture methods, *Escherichia* (*Escherichia coli*), *Enterococcus* (*Enterococcus cecorum*), and *Salmonella* (*Salmonella* spp.) were not overrepresented in BCO samples.

**Fig 9:**
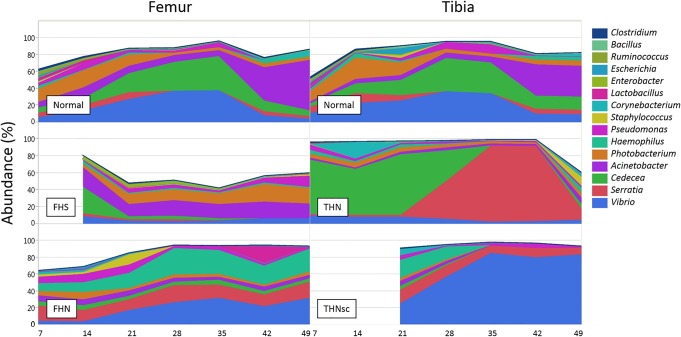
Taxonomic composition of the bone samples based at genus level. Only 15 major genera of significant importance were selected and shown in the graphs.

The community structure of femor associated bacteria differed significantly from tibial bacteria assemblages (Table 1). The observed difference may be due to the changes in physiological micro-environments caused by the different torque and shear stresses imposed on the femoral vs. tibial joints. For this analysis we selected 51 femur and 46 tibia samples (S1 Table). The results of taxonomic analysis of the V6 16S rRNA gene amplicon reads show that there are a total of 9 phyla across both bone locations, with the dominant phylum being *Proteobacteria* (mean 89.7% for the femur and 91.6% for the tibia) and followed by *Firmicutes* (6.8% for the femur and 5.7% for the tibia) and *Actinobacteria* (2.9% for the femur and 2.3% for the tibia), accounting for more 99% of all reads (Fig 4). We also compared the changes in bacterial communities in both femur and tibia at genus level. Fig 9 shows the distribution of major genera that belong to either *Proteobacteria* or *Firmicutes*. For the normal bones, there was a striking similarity in the changing patterns of the major genera over time between the femur and tibia. On the contrast, however, the development of the patterns was very distinctive between the femur and tibia with BCO lesions. It is interesting that the abundance of the genera *Serratia* and *Vibrio* increased to nearly 80% of total bacterial population at the later stages for THN and THNsc, respectively.

**Fig 10:**
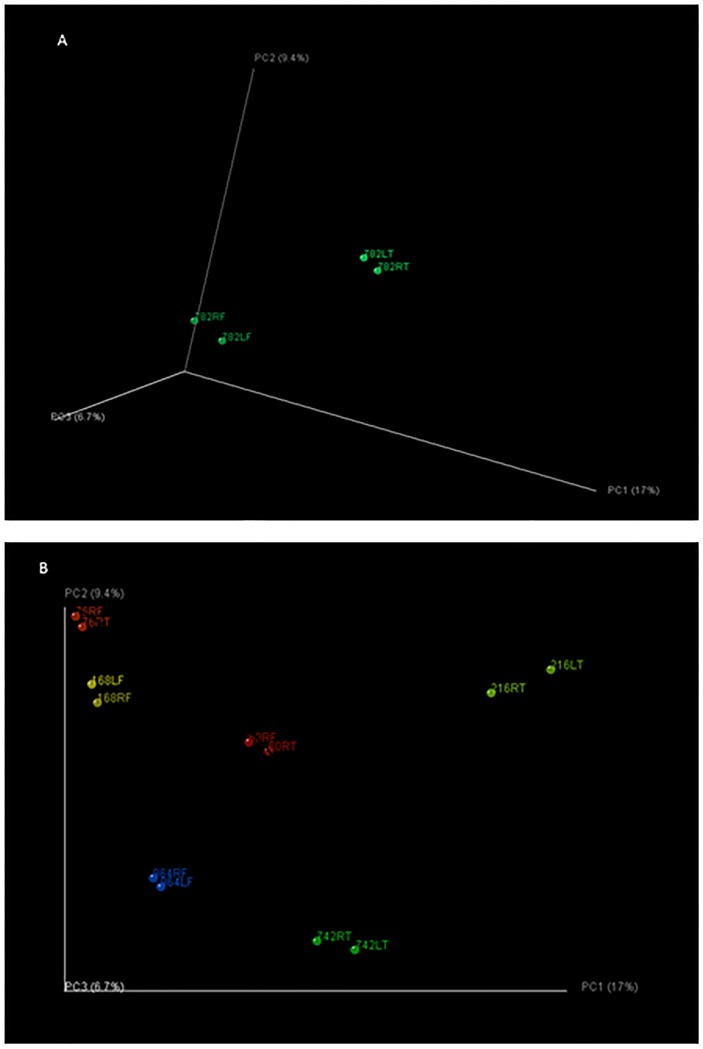
Comparison of samples using unweighted UniFrac. Unweighted UniFrac was used to generate a matrix of pairwise distances between communities, then a scatterplot was generated from the matrix of distances using Principal Coordinate Analysis. Number = chicken ID, R = right, L = left, F = femur, T = tibia. A. Comparison of 4 samples from the same chicken, which has FHS on both femurs and THN on both tibias. B. Comparison of the samples from different individual chickens. Paired samples from same individual chickens have FHS on femurs and THN on tibias.

ANOSIM analyses shows that individual chickens harbored distinctly different bacterial communities (Table 1). Recent research has demonstrated a high degree of inter-individual variability in the composition of bacterial communities at particular locations, such as human skin [59], and the chicken ceca [36]. Principal Coordinate Analysis showed that bacterial communities within the same bird are closely related, suggesting the presence of distinctive selective pressures in different individuals (Fig 10).

ANOSIM analyses showed that bacterial communities were similar among samples collected from birds reared on different flooring types (Table 1, litter vs. wire; p = 0.248). These data indicate that BCO triggered by wire flooring does not differ microbiologically from BCO that develops spontaneously in hatch-mates reared on wood shavings litter. This observation further validates the use of wire flooring model of BCO lameness to understand the etiology, pathogenesis, and treatment strategies for BCO lameness developed under normal rearing condition using a litter floor.

## Conclusions

Significant differences were observed between the bacterial communities of bone samples that appeared to be macroscopically normal versus samples having obvious BCO lesions (p≤0.001). The previously detected genus *Staphylococcus* was overrepresented in samples with BCO lesions. Two other genera overrepresented in the BCO samples, *Enterobacter* and *Serrotia*, might contribute to the pathogenesis of BCO. Interestingly, significant differences were also observed between the bacterial communities of the femora and tibiae, and among individual birds (p≤0.001). Our study illustrates that even macroscopically normal samples host complex bacterial communities. Taxonomic analysis shows that *Proteobacteria* (mean 90.9%) is the most abundant phylum, followed by *Firmicutes* (6.1%) and *Actinobacteria* (2.6%), accounting for more than 99% of all assembled reads. The dominant phylum *Firmicutes* in chicken cecal samples was shifted to *Proteobacteria* in chicken bone samples. Understanding the reasons responsible for this shift might be helpful for detecting the routes used by bacteria to infect the bones. Rarefaction curves demonstrated that α diversity decreased in general with increasing severity of BCO lesions in both femur and tibia. This observation suggests that unidentified selection processes favor the survival of certain bacterial subgroups in association with progression of BCO lesions. One limitation of the short-read sequencing technology used in this study is that it does not provide sufficient sequence information for the classification at species level. Thus, the majority of the sequences could be classified only at the genus level. On other hand, the potential bias of PCR and error in Illumina sequencing might have affected the linear relationship between the number of sequences per OTU and the different taxonomic groups of bacteria in the population. In future studies, longer regions (such as 16S rRNA V1V2 region), previously shown to be most variable within genus *Staphylococcus*, should be chosen to identify overrepresented bacteria in BCO samples at species level. To our knowledge, this is the first comprehensive survey of bacterial communities associated with BCO lesions in broilers using culture-independent method. The results provided various insights on the possible association between the observed changes in bacterial communities and BCO pathogenesis. The results of this study warrants further investigation of the bacterial communities of BCO lesions along with different types of samples such as cecal and blood samples from the same individual birds using the similar 16S rRNA gene profiling as well as quantitative PCR analysis to estimate bacterial loads.

## Supporting Information

S1 Table Summary of the samples used in this study. (XLSX)

S2 Table Design of PCR primers. (XLSX)

S3 Table Dominant taxonomic groups. (XLSX)

S4 Table Bacterial species isolated from the bones of the broilers with BCO. (XLSX)
